# Association between delayed cesarean section and severe maternal and adverse newborn outcomes in the Somaliland context: a cohort study in a national referral hospital

**DOI:** 10.1080/16549716.2023.2207862

**Published:** 2023-05-09

**Authors:** Jonah Kiruja, Fatumo Osman, Jama Ali Egal, Marie Klingberg-Allvin, Helena Litorp

**Affiliations:** a School of Health and Welfare, Dalarna University, Falun, Sweden; b School of Health and Welfare, University of Hargeisa, Hargeisa, Somaliland; c Department of Women’s and Children’s Health, Karolinska Institutet, Stockholm, Sweden; d Department of Women’s and Children’s Health, Uppsala University, Uppsala, Sweden; e Department of Global Public Health, Karolinska Institutet, Stockholm, Sweden

**Keywords:** Delayed caesarean section, severe maternal outcomes, adverse newborn outcomes, barriers, Somaliland

## Abstract

**Background:**

In a critical obstetric situation, the time interval between the decision of performing a caesarean section (CS) and delivery can influence maternal and newborn outcomes. In Somaliland, consent for surgical procedures, such as CS needs to be sought from family members.

**Objective:**

To determine the association between a delay in performing a CS and severe maternal and newborn outcomes in a national referral hospital in Somaliland. The type of barriers leading to delayed performance of CS after a doctor’s decision were also explored.

**Methods:**

Women were followed from the time of decision to perform CS until discharge from the hospital between 15 April 2019 and 30 March 2020. No delay was defined as < 1 hour and delayed CS was defined as 1–3 hours and >3 hours from decision of CS to delivery. Information was collected on barriers leading to delayed CS and maternal and newborn outcomes. Data was analysed using binary and multivariate logistic regression.

**Results:**

Overall, 1255 women were recruited from a larger cohort of 6658 women. A delay in CS >3 hours was associated with higher odds of severe maternal outcomes (aOR 1.58, 95% CI [1.13–2.21]). On the contrary, delay in performing a CS >3 hours was associated with lower odds of stillbirth (aOR 0.48, 95% CI [0.32–0.71]) compared to women without delay. Further, family decision-making for consent was the most important barrier leading to delays of >3 hours as compared to financial factors and barriers related to healthcare providers (48% vs 26% and 15%, respectively, *p* < 0.001).

**Conclusions:**

In this setting, delay in performing CS >3 hours was associated with higher risk of severe maternal outcomes. A standardised system of performing a CS by primarily addressing the barriers associated with family decision-making, financial aspects and healthcare providers is needed.

## Introduction

Although the global maternal mortality ratio (MMR) has declined significantly from 342 in 2000 to 211 in 2017 per 100,000 live births, the MMR in sub-Saharan Africa remains high at 542 per 100,000 live births [1]. Moreover, worldwide, over 2.65 million foetal deaths occur annually, with 75% of these foetal deaths taking place in sub-Saharan Africa and South Asia [2]. In Somaliland, the MMR of 732 per 100,000 live births and a neonatal mortality ratio of 39 per 1,000 live births makes the country one of the most affected in the world [3]. To prevent severe maternal and adverse newborn outcomes, a caesarean section (CS) is recommended as a vital intervention that can prevent severe maternal outcomes (maternal near-miss or maternal mortality) and newborn mortality at birth. Maternal near miss is when a woman develops severe obstetric complications and almost dies but ultimately survives during pregnancy, delivery, or within six weeks postpartum [4]. Thus, a CS is an essential intervention in cases of obstructed labour, foetal distress, and other obstetric emergencies. Despite scaling up the capacity of medical facilities to perform CSs in all regions of Somaliland [5], the national CS rate in Somaliland remains low at 4% [6], thereby indicating a possible underuse of the procedure that might have implications for maternal and newborn health outcomes.

Previous studies have described that a delay in performing a CS has a negative effect on maternal and foetal outcomes in many cases [7–9]; however, there are also contradictory findings that indicate that a prolonged decision-to-delivery time interval does not affect foetal-maternal outcomes [10,11]. These differences might be explained by maternal-foetal conditions, such as ruptured uterus and foetal distress, that pose an immediate threat to the woman or foetus and, thus, requiring a CS without delay [7–9]; on the other hand, other complications, like cephalopelvic disproportion, are less time-sensitive [10,11]. Further, previous studies have identified factors related to healthcare providers – such as poor labour and foetal monitoring, delayed action in managing obstetric complications, lack of money for hospital expenses, and lack of knowledge regarding danger signs – as factors that contribute to a delay in performing a CS (hereafter referred to as ‘CS delay’ for brevity) [11]. Further, a previous study conducted in Somaliland revealed that an emergency CS cannot be performed by healthcare providers until required consent is provided by the pregnant woman’s extended family [12].

When CS is indicated in Somaliland, a male family member, often the husband or the father to the woman undergoing CS or both, are required to give consent for CS to be performed. A male blood relative can also give consent for the woman to undergo CS [12]. The decision-making process is, however, often undertaken in considerable deliberation among family members and waiting for the husband or male family member to arrive to the hospital to give consent can be time consuming [12]. In another study conducted in Somaliland, obstetric health care providers indicated this aspect to be a major contributing factor to delayed obstetric interventions and increased burden of adverse maternal outcomes [13]. However, factors associated with CS delay in Somaliland and the effect of CS delay on maternal and newborn outcomes have not been investigated thus far. Therefore, the primary objective of this study was to determine the association between CS delay and severe maternal and newborn outcomes. The secondary objective was to explore what type of barriers lead to delayed performance of CS after a doctor’s decision.

## Materials and methods

### Study design

For the current study, we used a prospective cohort study design nested in a larger maternal near-miss (MNM) study (*n* = 6658) reported elsewhere [14]. The study was reported in accordance with the Strengthening of Reporting of Observational Studies in Epidemiology (STROBE) guidelines of cohort studies [15]. Ethical clearance was obtained by the Somaliland Ministry of Health Development (MOHD/DG: 2/165/2018) and University of Hargeisa (Dr: CS/41105/18).

### Study setting

The study site was the obstetrics department in the main national referral hospital (Hargeisa Group Hospital (HGH)) in Hargeisa, Somaliland, a low-income country in the Horn of Africa, with vast healthcare system and socio-economic challenges [3]. In Somaliland, CSs are mainly performed within the public healthcare system [16]. Approximately 6000 deliveries are performed at HGH, with an estimated 1,000 CSs per year. Comprehensive emergency obstetric care is provided by approximately 40 staff, comprising senior and junior consultant obstetricians, residents, junior doctors, nurses, and midwives. The hospital has anaesthetists, a blood transfusion centre, a clinical laboratory, and an intensive care unit. Nurses/midwives perform initial health assessments of pregnant women. Doctors then triage women and perform further assessments, such as ultrasound and laboratory investigations as a foundation for the decision to resort to a CS. In Somaliland, either the husband or father of the woman giving birth have to provide written consent provided in writing or a thumbprint for emergency obstetric interventions. If no such male relatives are available, the senior medical doctor in consultation with the hospital administration can authorise a CS [12,13,17].

### Study population

All women with a singleton pregnancy who underwent a CS at the study site between 15 April 2019 and 30 March 2020 were included in the study. We excluded women for whom a decision to perform a CS was made but the woman did not undergo the procedure or died before a CS could be performed. Similarly, women with missing information on the variables related to a delay in performing a CS were excluded from the study.

### Role of the authors

Authors in this study were not employed at the study site or involved with provision of care to women that underwent CS. However, the authors involved in this study are healthcare professionals comprising registered nurses, midwives and an obstetrician with obstetrics experience in low-resource settings.

### Data collection

Using the modified World Health Organisation Maternal Near Miss (WHO MNM) tool, we collected prospective data on all women admitted to the obstetric department for childbirth from 15 April 2019 to 30 March 2020 [14]. The data abstraction form was adapted from the WHO MNM tool to also include criteria in accordance with the more recently developed sub-Saharan Africa (SSA) near-miss criteria, which includes additional inclusion criteria for low-resource settings with the purpose of averting underreporting of severe maternal morbidity due to limited resources [4,18]. The instrument was further discussed in a reference group, comprising of local healthcare providers involved in the care, as to contextualise the main variables contributing to delayed performance of CS. The data collection instrument was attached to each pregnant woman’s medical record at admission. The assigned midwife extracted data from the woman’s medical record to the data collection instrument in each shift. The completed tools were also double checked against the admission and discharge information system in the hospital. The completed data collection instruments were regularly checked by JE and JK to ensure accuracy and completeness.

Data were entered into the Statistical Package of Social Sciences (SPSS) version 22 by JE and JK, who were assisted by trained data entry persons. Interim analysis was conducted quarterly to systematically check for missing data and data quality. This provided the opportunity to discuss various means of improving data quality with those who collected the data. Missing or incorrect data were rectified by reviewing the data abstraction forms and the woman’s medical records.

### Study variables

#### Exposure variable

The exposure variable for the current study was CS delay, which was defined as the time interval from decision made by the medical doctor to perform a CS to the actual delivery of the baby. Data on the exposure variable was collected in categories of ‘less than 15 minutes’, ‘15 to 59 minutes’, ‘1 hour to 2 hours and 59 minutes’, and ‘3 hours and over’. For the current study, we explored delays in three different categories: (1) no CS delay (defined as CS being performed within one hour after decision), (2) a CS delay of between one and three hours, and (3) a CS delay of over three hours. Although acceptable delay of CS depends on the indication [19], many low-resource settings struggle with provision of timely CS. The current study strived to take such contextual factors into account. Before starting the current study, a project meeting was held that included nurses, midwives, and doctors at the study site based on their expert opinions, experiences, and with consideration of the local context. The cut-offs for CS delays used in this study were then agreed upon and are also in line with the recommended ranges in the extant literature on CS to be performed within 30 minutes to 3 hours depending on the indication of the operation [19].

#### Outcome variables

We explored a delay in the performance of CS in relation to two outcomes related to maternal vs. newborn health. First, we explored the impact of a delay in the performance of CS on severe maternal outcomes (SMO) defined as maternal death according to the WHO definition or maternal near-miss based on the SSA maternal near-miss criteria [18]. Such maternal near-miss events include cardiovascular, respiratory, renal, coagulation, hepatic, neurologic, and uterine dysfunction – as defined by the WHO – as well as women with severe preeclampsia with ICU admission, eclampsia, sepsis or severe systemic infections, pulmonary oedema, transfusion of ≥ 2 units of red blood cells, and uterine rupture [18]. Second, we explored the impact of CS delay on newborn outcome, defined as the baby being born alive or being stillborn, as registered in the medical record. We also investigated the role of family decision-making, financial factors, and barriers related to healthcare providers as reasons leading to a delay in the performance of a CS. In this study, a delay in family decision-making was defined as a time-consuming process caused by family members when consulting to finally decide and give permission for a CS to be performed. A delay due to financial factors was defined as a delay in the performance of CS caused by lack of the family’s ability to pay for the costs of a CS. The delay caused by healthcare providers was defined as the poor quality of care provided by healthcare providers – such as nurses, midwives, or doctors – that contributes to delayed CS.

#### Co-variables

Co-variables included in the analyses as potential confounding factors were obstetric factors associated with a CS, such as ruptured uterus, eclampsia, severe antepartum haemorrhage, as well as sociodemographic factors including maternal age, education, residence, gestational age, parity, and mode of referral.

### Statistical analyses

Using SPSS version 22, data were analysed using descriptive statistics to present the sociodemographic and obstetric characteristics of study participants. Barriers leading to delayed CS and the underlying cause for CS was explored among women with and without a one-hour delay and three-hour delay in the performance of CS, respectively, using descriptive statistics, including the chi-square test. Statistical significance was set at *p* < 0.05. Further, we conducted bivariate and multivariate analyses to calculate the crude and adjusted odds ratios (cOR and aOR), including 95% confidence intervals (CI), to assess whether a delay in the performance of a CS increased the odds of adverse maternal and foetal outcomes compared to no delay in the performance of CS in terms of maternal outcome (SMO vs. no SMO) and newborn outcome (stillborn or alive). In addition, we adjusted for maternal age, education, and parity.

## Results

**Figure 1.**
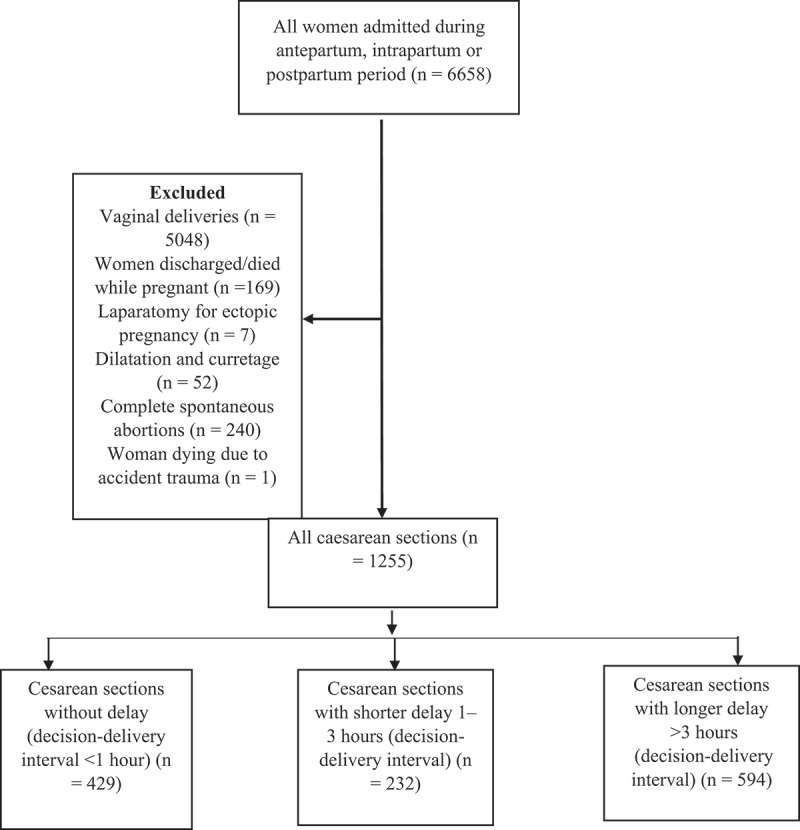
Flow chart of caesarean sections included in this study from the original maternal near-miss study.

From the original maternal near-miss study including 6,658 women [14], 1255 women underwent a CS and were included in the analyses (Figure 1). Among the total number of women included, 429 (34.2%) did not experience CS delay, 237 (18.9%) experienced CS delay ranging from one hour to less than three hours, and 594 (43.3%) experienced CS delay of three hours and over.

### Sociodemographic characteristics of study participants

Nulliparous women were more common in the 1–3 hour and >3 hours CS delay groups than in the group without CS delay (21.1% and 20.9% vs 10.0%, respectively, *p* < 0.001) (Table 1). A larger proportion of women with no formal education was found in the >3 hours CS delay than in the groups with shorter or no delay (64.6% vs 53.0% and 55.5%, *p* = 0.007). Further, referral from other healthcare providers was more common in the group without delay than in the 1–3 hours and >3 hours CS delay groups (15.4% vs 2.2% and 4%, respectively, *p* < 0.001). No significant difference was observed between the two groups in terms of place of residence (*p =* 0.124).

**Table 1.** Background characteristics of study participants for studying the outcomes of CS delay.

	No CS delay n = 429 (%)	CS delay one – three hours n = 232 (%)	CS delay >three hours N = 594 (%)	p-value
***Maternal Age***				
<20 yrs	55 (12.8%)	41 (17.7%)	105 (17.7%)	0.036
20–34 yrs	297 (69.2%)	410 (70.3%)	378 (63.6%)	
≥35 yrs	77 (17.9%)	28 (12.1%)	111 (18.7%)	
Missing	0 (0.0%)	0 (0.0%)	0 (0.0%)	
Total	429 (100.0%)	232 (100.0%)	594 (100.0%)	
***Parity***				
0 (Nulliparous)	43 (10.0%)	49 (21.1%)	124 (20.9%)	<0.001
1–4 (Multiparaous)	292 (68.1%)	162 (69.8%)	384 (64.6%)	
>4 (Multiparous)	94 (21.9%)	21 (9.1%)	86 (14.5%)	
Missing	0 (0.0%)	0 (0.0%)	0 (0.0%)	
Total	429 (100.0%)	232 (100.0%)	594 (100.0%)	
**Previous caesarean^a^** **section (among multiparous)**				
Yes	232 (60.1%)	149 (81.4%)	296 (63.0%)	<0.001
No	154 (39.9%)	34 (18.6%)	174 (37.0%)	
Missing	0 (0.0%)	0 (0.0%)	0 (0.0%)	
Total	386 (100.0%)	183 (100.0%)	470 (100.0%)	
**Level of Education**				
Non-formal Education	238 (55.5%)	123 (53.0%)	384 (64.6%)	0.007
Primary and Secondary School	187 (43.6%)	108 (46.6%)	207 (34.8%)	
University	4 (0.9%)	1 (0.4%)	3 (0.5%)	
Missing	0 (0.0%)	0 (0.0%)	0 (0.0%)	
Total	429 (100.0%)	232 (100.0%)	594 (100.0%)	
**Place of residence**				
Rural	35 (8.2%)	16 (6.9%)	65 (10.9%)	0.124
Urban	394 (91.8%)	216 (93.1%)	529 (89.1%)	
Missing	0 (0.0%)	0 (0.0%)		
Total	429 (100.0%)	232 (100.0%)	594 (100.0%)	
**Referral status**				
Traditional birth attendant	4 (0.9%)	1 (0.4%)	10 (1.7)	<0.001
Healthcare providers at maternal and child health centres	66 (15.4%)	5 (2.2%)	24 (4.0%)	
Self-referred	359 (83.7%)	226 (97.4%)	560 (94.2%)	
Missing	0 (0.0%)	0 (0.0%)	0 (0.0%)	
Total	429 (100.0%)	232 (100.0%)	594 (100.0%)	

^a^Nulliparous, not included in the analysis.

### Association between delayed CS and type of barrier leading to delayed decision to perform CS

In the analysis of type of barrier leading to delays in performing CS, 429 cases were excluded since there was no CS delay and, hence, no registered reason. In the group with a CS delay of 1–3 hours, financial barriers causing a delayed provision of consent was the most common barrier to the performance of CS as compared to family decision-making and barriers related to healthcare providers (40.1% vs 33.2% and 26.7%) (Table 2). However, family decision-making was the most common barrier leading to delayed CS in the >3 hours CS delay group (48.3%; *p* < 0.001).

**Table 2.** Association between delayed CS and type of barrier to delayed CS.

	CS delay 1–3 hours *n* = 232 (%)	CS delay >3 hours *n* = 594 (%)	p-value
**Barriers to consent**^**a**^			
Healthcare providers barriers	62 (26.7%)	91 (15.3%)	<0.001
No healthcare provider barrier	170 (73.3%)	503 (84.7%)	
**Total**	**232 (100.0%)**	**594 (100.0%)**	
Financial barriers	93 (40.1%)	216 (36.4%)	<0.001
No Financial barriers	139 (59.9%)	378 (63.6%)	
**Total**	**232 (100.0%)**	**594 (100.0%)**	
Family decision-making barriers	77 (33.2%)	287 (48.3%)	<0.001
No family decision-making barriers	155 (66.8%)	307 (51.7%)	
**Total**	**232 (100.0%)**	**594 (100.0%)**	

^a^There were *1255* caesarean section cases; however, *429* caesarean section cases were excluded since they was no CS delay (<1 hour)

### Association between CS delay and the underlying cause of SMO

As presented in Table 3, obstetric haemorrhage was a more common cause for SMOs among women with >3 hours delay in obtaining consent for performing a CS as compared to women without delay or with 1–3 hours delay of obtaining consent to perform CS (43.3% vs 15% and 6.7%, respectively, *p* < 0.001). Further, hypertensive disorders were more common among women with no CS delay compared to women with >3 hours and 1–3 hours CS delay (30% vs 27.8% and 10%, respectively); however, the results were not statistically significant (*p* = 0.1039). Severe anaemia was most common among women with CS delay of 1–3 hours of delay than in the groups with no delay and the group with >3 hours CS delay (83.3% vs 52.5% and 19.6%, respectively). The result was statistically significant (*p* < 0.001).

**Table 3.** Association between delayed CS and underlying causes of severe maternal outcomes.

	No CS delay n = 40 (%)	CS delay 1–3 hours n = 30 (%)	CS delay >3 hours n = 97 (%)	p-value
***Underlying causes of severe maternal outcomes***^***a***^				
Obstetric haemorrhage	6 (15.0%)	2 (6.7%)	42 (43.3.0%)	<0.001
Hypertensive disorders	12 (30.0%)	3 (10.0%)	27 (27.8%)	0.1039
Pregnancy-related infection	1 (2.5%)	0 (0.0%)	7 (7.2%)	0.3187
Severe anaemia	21 (52.5%)	25 (83.3%)	19 (19.6%)	<0.001
Medical complications^b^	0 (0.0%)	0 (0.0)	2 (2.1%)	1.0
Missing	0 (0.0%)	0 (0.0%)	0 (0.0%)	

^a^The total number of underlying causes of severe maternal outcomes were less than 1255, as only 158 cases of maternal near-miss according to sub-Saharan African criteria and 9 maternal deaths were included in the analysis.

^b^medical complications diagnosis = pneumonia (2).

### Association between CS delay and maternal and newborn outcomes

The total number of maternal deaths were 9 while maternal near miss were 158. A lower proportion of women without CS delay (9.3%) suffered from SMO as compared to women with 1–3 hours CS delay (12.9%) and women with >3 hours CS delay (16.3%, *p* = 0.005) (Table 4). On the contrary, neonates had lower mortality (6.6%) in the group of women with >3 hour CS delay as compared to women without delay and 1–3 hours CS delay (12.1% and 12.5%, respectively; *p* = 0.003).

**Table 4.** Delayed CS and proportion of severe maternal outcomes and newborn death at birth in the respective groups.

	No CS delay n = 429 (%)	CS delay 1–3 hours *n* = 232 (%)	CS delay >3 hours *n* = 594 (%)	*p*-value
***Maternal outcome***				
No Severe maternal outcome	389 (90.7%)	202 (87.1%)	497 (83.7%)	0.005
Severe maternal outcome^a^	40 (9.3%)	30 (12.9%)	97 (16.3%)	
Missing	0 (0.0%)	0 (0.0%)	0 (0.0%)	
Total	429 (100.0%)	232 (100.0%)	594 (100.0%)	
***Newborn outcome at birth***				
Alive	377 (87.9%)	203 (87.5%)	555 (93.4%)	0.003
Stillborn	52 (12.1%)	29 (12.5%)	39 (6.6%)	
Missing	0 (0.0%)	0 (0.0%)	0 (0.0%)	
Total	429 (100.0%)	232 (100.0%)	594 (100.0%)	

^a^Severe maternal outcomes comprised 9 maternal deaths and 158 maternal near miss. In the group with no CS delay there were 2 maternal deaths and 38 maternal near miss, in the group with CS delay of 1–3 hours 2 maternal deaths and 28 maternal near miss and in the group with CS delay > 3 hours 5 maternal deaths and 92 maternal near miss.

After adjustment for maternal age, maternal education, and parity, we found that women with a CS delay of >3 hours had 1.58 times higher odds (aOR 1.58, 95% CI 1.13–2.21) of SMO than women without delay in obtaining consent for performing a CS (Table 5). On the contrary, women with a CS delay >3 hours had lower risk of stillbirth than women without CS delay (aOR 0.48, 95% CI 0.32–0.71). In addition, no statistically significant association was found between 1 and 3 hours CS delay and SMO and stillbirth (aOR 1.50, 95% CI 0.89–2.50 and aOR 1.00, 95% CI 0.61–1.64, respectively).

**Table 5.** Bivariate and multivariate logistic regression analyses with crude and adjusted odds ratios (OR) of severe maternal outcome stillbirth with CS delay of 1–3 hours and >3 hours.

	Crude OR (95% CI)			Adjusted OR* (95% CI)		
Outcome	No CS delay	CS delay of 1–3 hours	CS delay >3 hours	No CS delay	CS delay of 1–3 hours	CS delay >3 hours
Severe maternal outcome	1.0	1.04 (0.64–1.68)	1.65 (1.19–2.30)	1.0	1.50 (0.89–2.50)	1.58 (1.13–2.21)
Newborn outcome died at birth	1.0	1.44 (0.87–2.39)	0.50 (0.34–0.75)	1.0	1.00 (0.61–1.64)	0.48 (0.32–0.71)

*adjusted for maternal age, maternal education, and parity.

## Discussion

To the best of our knowledge, this study is the first to demonstrate an association between CS delay and severe maternal outcomes. Review of related literature on the association between CS delay and severe maternal outcomes yielded no study that has used the sub-Saharan Africa criteria to define the severe maternal outcome of maternal near miss. Our study provides novel insight on how delayed CS contributes to severe maternal outcomes defined as both maternal deaths and maternal near miss according to the sub-Saharan Africa criteria [18]. We also identified family decision-making and financial barriers as the most important contributing factors to a delay in the performing a CS >3 hours and 1–3 hours, respectively. However, contrary to our earlier understanding, a longer CS delay of >3 hours was associated with a lower risk for stillbirth as compared to no delay or shorter delay (1–3 hours).

Our study reports a high proportion of CS delay in this study setting and also reveals that nulliparous women were more common in the groups with CS delay as compared to the group without delay. As revealed in previous studies, nulliparous women are at a higher risk than multiparous women with pregnancy complications, such as preeclampsia/eclampsia and preterm labour, both of which contribute to severe maternal outcomes [20,21]. Women with no formal education were also more common in the groups with a delay in obtaining consent for performing a CS, thereby confirming findings from other studies that low levels of education are associated with delayed acceptance of recommended emergency obstetric care and, possibly, substandard care [9,22]. Most of the women referred by other healthcare providers provided consent without delays (15.4% vs 2.2% and 4.4% in 1–3 hours and >3 hours, respectively) as compared to women who referred themselves directly to the hospital (83.7% vs. 97.4% and 94.2% in 1–3 hours and >3 hours, respectively) as well as those referred by traditional birth attendants (0.9% vs 0.4% and 1.7% in 1–3 hours and >3 hours, respectively). It is possible that care was provided more promptly to this group due to referral and the medical indication was more urgent. This finding reveals that it is possible that the initial interaction women and family members had with healthcare providers being admitted to primary healthcare facilities influenced them to provide consent for CS without delay at the referral hospital [22]. In summary, our findings are consistent with previous literature that indicates that obstetric and sociodemographic factors among women who undergo a CS can play an important role in the process of obtaining consent for emergency situations [11,22,23]. These factors can influence how consent for performing a CS is sought and understood and can contribute towards delays, which is in contrast to a timely provision of consent for performing a CS.

In the >3 hours CS delay group, the incidence of family decision-making as a barrier leading to delay in performing CS was higher than other barriers that were analysed in this study. This finding is consistent with findings from a cross-sectional study on the implications of informed consent practices on emergency obstetric care conducted in Nigeria [24]. In the Nigerian study, consent was more likely to be delayed when it was given by husbands (*p* = 0.019) or relatives (*p* = 0.004). Previous studies have indicated that the practice of relying on male partners or family members to provide surgical consent for women is prevalent in rural populations, women from low-income families, and among women with low levels of education [11,22–24]; hence, our study provides further evidence of the importance of health literacy and financial systems in low-income settings as strategies to improve the use of emergency obstetric care [25,26]. The provision of antenatal care in Somaliland is low at 47% [6], thereby indicating the need for improvements in the healthcare system and building women’s trust in the healthcare provided to them. Information regarding provision of consent during emergencies can be part of antenatal care. In the context of related policies in Somaliland, the requirement of family consent for surgical interventions has been the subject of debate among service providers, policymakers, and healthcare associations over the last decade [27]. Discussions among these groups on drafting and enacting a policy on maternal health emergencies are still ongoing.

Our study clearly demonstrates how delayed CS increases the risk of severe maternal outcomes, with >3 hours CS delay resulting in a higher risk of severe maternal outcomes as compared to 1–3 hours delay of CS. This finding is consistent with evidence from similar settings that reveal that the interval between the delay in CS decision to delivery time contributed to severe acute maternal morbidity and maternal deaths [11,22]. According to previous studies, possible reasons that can contribute to the delay of obtaining consent for CS include delays in patient preoperative preparation, delays in the preparation of surgical kits, delays in the transfer of the patient from the ward to the operating theatre, delays in being able to pay the required surgical fees, lack of money to buy surgical kits, and delayed decision-making on the part of families [22,27]. Healthcare providers need to be trained to have adequate skills on how to communicate and effectively seek consent from their patients and family members and motivate them to make a prompt decision when there is a need for an emergency procedure such as a CS [28]. Our study highlights that interventions related to improving the quality of the informed consent process, as implemented in other contexts with delayed CS [29], would facilitate a shorter interval between decision to perform a CS to delivery time, thereby reducing adverse maternal and newborn outcomes. Such interventions might target comprehensive emergency obstetric care health facilities and healthcare providers and include the development of a standardised informed consent protocol. The protocol can entail components such as indication for procedure, discussion of associated risks, implications for future pregnancies, verbal and written consent enquiry, and opportunity for family members to ask questions. Another intervention can involve training healthcare providers who provide antenatal care on how to prepare pregnant women and their family members/husbands who are responsible for providing consent to be ready to make a decision for performing a CS and be well-informed about all the aspects involved [29].

Contrary to our hypothesis, the current study revealed a reduced risk of stillbirth in the >3 hours CS delay group. However, our finding corroborated the findings from other studies that indicated that the period of CS delay from decision to delivery at 3 hours and above did not contribute to stillbirths [11,30–32]. One possible explanation for this might be that CSs with longer delays had additional maternal indications other than foetal distress, and that CSs in which there was risk of foetal compromise were performed with more urgency. Furthermore, it is also possible that our results are biased by an underreporting of newborns with severe acute morbidity, as we did not collect data on other newborn outcomes, such as low APGAR scores, mask ventilation and or/newborn resuscitation, and transfer to the newborn intensive care unit. Moreover, we did not follow up newborns after birth – it is possible that newborns with severe morbidity were born alive but died subsequently, before discharge from hospital. However, the data collection we did allowed for analyses of newborn mortality.

### Strengths and limitations of the study

One of the major strengths of this cohort study was that the first author regularly identified and eliminated missing data, thereby ensuring data quality. Given that the study setting was the national referral hospital, which provides emergency obstetric care to women from all regions of Somaliland, the study population is representative of women living in Somaliland in terms of demographic and socioeconomic characteristics. However, a limitation of our study is that neonates who were born alive but with severe morbidity and/or died were not identified. Therefore, it is possible that our results might be biased in terms of newborn outcome analysis by an underreporting of newborns with acute morbidity risk, as we did not collect data on other newborn outcomes, such as low APGAR scores, mask ventilation and/or newborn resuscitation, and transfer to the newborn intensive care unit. Moreover, as the main study was not designed to collect data specifically on women who were recommended CS but never underwent the procedure due to unexpected spontaneous vaginal delivery, detection of absent foetal heart rate or maternal death while waiting for surgery, these women were not included in the study. Analysing such women would have added valuable information on the impact of delay on maternal and perinatal outcomes. It is possible that exclusion of these groups could explain under reporting of the number of newborns with the outcome of stillbirth, which resulted to the odd finding of reduced risk of stillbirth with >3 hours of CS delay. Information about women’s ANC attendance would further have been valuable. The confounders included in the multivariate analysis were based on previous research and clinical experience, but we cannot rule out that one or more other important confounders were not included during the multivariate analysis. Our findings are not only relevant for Somaliland context but other low resource settings as well. However, although our findings might be generalised to contexts with similar sociocultural and demographic characteristics, it may not be possible to generalise them to contexts in which consent for life-saving interventions such as performing a CS is provided by the woman herself.

## Conclusion

This study found that an increased delay in performing a CS was associated with increased risk of SMO and that healthcare providers, financial factors, and family decision-making were important barriers that contributed to a delay in the performance of CS. Our findings call for the need of establishing a standardised system of addressing the identified barriers that cause a delay in performing a CS – for example, by training healthcare providers in obtaining consent and using associated protocols and guidelines to ease the decision-making process. The antenatal care period can also be utilised to obtain informed consent from the pregnant woman and her family early during antenatal care visits in order to avoid unnecessary morbidity and mortality among women who experience complications during delivery. This study suggests that women’s experiences, views and perspectives on getting CS consent from family members be explored in further studies.

## Paper context

As compared to previous research on CS delay and various obstetric diagnoses, this study analysed the association of CS delay with life-threatening conditions defined as severe maternal outcomes according to the sub-Saharan African near-miss criteria. We found that prolonged CS delay >3 hours contributed to SMOs. Our study adds new knowledge with regard to how delayed CS due to contextual factors, such as healthcare providers, financial factors, or family decision-making leading to delayed CS contribute to SMOs in Somaliland.
